# TRAJECTORIES OF SOCIAL SUPPORT AMONG FAMILY CAREGIVERS: THE ROLE OF PSYCHOLOGICAL RESILIENCE

**DOI:** 10.1093/geroni/igae098.0640

**Published:** 2024-12-31

**Authors:** Jeremy Lim-Soh, Yongjing Ping, Ha-Linh Quach, Shannon Ang, Rahul Malhotra

**Affiliations:** Duke-NUS Medical School, Singapore, Singapore; Duke-NUS Medical School, Singapore, Singapore; Duke-NUS CARE, Singapore, Singapore; Nanyang Technological University, Singapore, Singapore; Duke-NUS Medical School, Singapore, Singapore

## Abstract

Social support has been widely acknowledged as an important factor impacting family caregivers’ outcomes including burden and depression. However, research focused on social support as an independent factor, and how it changes over time, is limited. This study is the first to describe heterogeneous trajectories (patterns of change over time) in perceived social support among family caregivers of older adults. It also investigates if caregiver psychological resilience influences these trajectories. This study draws on a longitudinal survey of 278 Singaporean family caregivers of community-dwelling older adults aged 75 years and above with activities of daily living limitations. Caregivers were interviewed up to four times across two years. Perceived social support and psychological resilience were measured by Pearlin’s and Connor-Davidson’s scales respectively. We applied Group-Based Trajectory Modeling, an approach that is sensitive to within-population heterogeneity, to describe change over time in perceived social support. We modeled psychological resilience as a time-varying covariate in the model. Five distinct trajectories of perceived social support were identified, with three stable trajectories (91% of caregivers) and two declining trajectories (9%). Psychological resilience was positively associated with perceived social support only in the stable trajectories. While most family caregivers of older adults may experience stable perceived social support over time, a minority do report declining perceived social support. Further investigation is needed to understand the reasons for this decline and design effective interventions targeting this vulnerable group.

